# Overall adjustment acupuncture improves osteoporosis and exerts an endocrine-modulating effect in ovariectomized rats

**DOI:** 10.3389/fendo.2022.1074516

**Published:** 2022-11-17

**Authors:** Xiang Li, Kenan Wu, Qinzuo Dong, Hongxi Chen, Chunyan Li, Zeqin Ren, Fan Liu, Xianwu Yue, Chunlin Xia, Yuanfeng Wang, Yingjing Luo, Li Li, Rong Zhao, Zuhong Wang, Dongdong Qin

**Affiliations:** ^1^ The First Clinical Medical School, Yunnan University of Chinese Medicine, Kunming, Yunnan, China; ^2^ The Second Clinical Medical School, Yunnan University of Chinese Medicine, Kunming, Yunnan, China; ^3^ Department of Life Technology Teaching and Research, School of Life Science, Southwest Forestry University, Kunming, Yunnan, China; ^4^ Department of Rehabilitation, The First Affiliated Hospital of Dali University, Dali University, Dali, Yunnan, China; ^5^ Department of Acupuncture, Qujing Hospital of Traditional Chinese Medicine, Qujing, Yunnan, China; ^6^ The Affiliated Hospital, Yunnan Institute of Traditional Chinese Medicine, Kunming, Yunnan, China; ^7^ Department of Acupuncture and Rehabilitation, Jingzhou Hospital of Traditional Chinese Medicine, Jingzhou, Hubei, China; ^8^ Department of Acupuncture, Kunming Hospital of Traditional Chinese Medicine, Kunming, Yunnan, China; ^9^ School of Basic Medical Sciences, Yunnan University of Chinese Medicine, Kunming, Yunnan, China

**Keywords:** overall adjustment acupuncture, postmenopausal osteoporosis, estradiol, hypothalamus-pituitary-adrenal axis, ovariectomy

## Abstract

**Background:**

Acupuncture is a widely practiced, convenient, and safe treatment modality within complementary and integrative medicine. Increasing studies have revealed the efficacy of acupuncture for the treatment of osteoporosis in both human and non-human subjects. The aim of the present study was to assess the improvement of osteoporosis after overall adjustment acupuncture (OA) as well as its endocrine-modulating effect in an ovariectomized rat model.

**Methods:**

In total, 32 female Sprague–Dawley (SD) rats were randomly divided into the sham, model, ovariectomy+estrogen (OVX+E), and OVX+OA (OVX+A) groups with eight rats in each group. The postmenopausal osteoporosis (PMOP) rat model was induced by bilateral ovariectomy. At 12 weeks after surgery, rats in the OVX+E group received estradiol (0.2 mg/kg/i.g./qod) for 12 weeks, and rats in the OVX+A group were treated with acupuncture at Zusanli (ST36), Shenshu (BL23), and Dazhu (BL11) points (qod) for 12 weeks. At the end of the treatment, all rats were sacrificed, and the body weight, uterus index, bone mineral density (BMD), bone mineral content (BMC), bone trabeculae structural parameters, femoral biomechanical properties, femoral histomorphology, and several hormone levels were examined.

**Results:**

In OVX rats, OA abrogated the body weight gain and improved osteoporosis in terms of BMD, BMC, bone trabeculae structural parameters, bone strength, and bone tissue histomorphology. Moreover, OA modulated the serum levels of estradiol, corticotropin releasing hormone (CRH), adrenocorticotropic hormone (ACTH), and corticosterone (CORT).

**Conclusions:**

OA improves osteoporosis and exerts an endocrine-modulating effect in ovariectomized rats.

## Introduction

Osteoporosis is a metabolic bone disorder that is characterized by low bone mass and microarchitectural deterioration of bone tissue with a consequent increase in bone fragility and susceptibility to fracture (1). A recent study has reported that the prevalence of osteoporosis among those aged 40 years or older is 5.0% among men and 20.6% among women in mainland China (2). Osteoporosis-related fractures are associated with increased disability, increased mortality, and low quality of life (3). Due to population aging, osteoporosis has become a major public health issue globally (4, 5).

Postmenopausal osteoporosis (PMOP), resulting from estrogen deficiency, is the most common type of osteoporosis (6). Estrogen deficiency increases bone turnover with an imbalance between bone formation and resorption, and it is the main cause of osteoporosis in women aged 50 years or older (7, 8). An active role of the immune system as well as increased production and release of a variety of cytokines in the bone marrow environment, such as granulocyte-macrophage colony stimulating factor (GM-CSF), tumor necrosis factor-alpha (TNF-alpha), interleukin-1, interleukin-6, and receptor activator nuclear kappa-b ligand (RANKL), are implicated in sex hormone deficiency-induced bone loss, which stimulates bone resorption by increasing the number and/or the activity of bone-resorbing osteoclasts (9, 10).

Several drugs are licensed to reduce fracture risk by slowing down bone resorption (such as bisphosphonates and denosumab) or by stimulating bone formation (such as teriparatide, abaloparatide, or romosozumab) (11). Moreover, menopausal hormone therapy (MHT) has also been demonstrated to be effective for the maintenance of skeletal health and prevention of future fractures, and the application of MHT is confined to women who have recently (<10 years) become menopausal, who have menopausal symptoms, and who are less than 60 years old with a low baseline risk for adverse events (12, 13). Given the adverse effects of prolonged estrogen therapy, including an increased risk of cardiovascular events, thromboembolic disease, and breast cancer, as well as the rare but potentially serious side effects of common osteoporosis medications (bisphosphonates, raloxifene, denosumab, and teriparatide) (14, 15), there is an urgent need for alternative treatments to increase bone mineral density (BMD) and prevent fractures with a good safety profile.

Acupuncture has a long history in traditional Chinese medicine (TCM) and has gained increasing acceptance and popularity worldwide in recent decades. With increasing evidence of its clinical efficacy, acupuncture is now a widely practiced convenient and safe treatment modality within complementary and integrative medicine worldwide (16). Application of acupuncture in the treatment of osteoporosis was described in Huangdi Neijing, the ancient classical text of acupuncture. In recent years, many studies have investigated the efficacy of acupuncture for the treatment of primary osteoporosis in clinical practice, and the findings have shown that acupuncture is effective in improving osteoporosis (17–20). Moreover, a previous study on secondary osteoporosis in patients with spinal cord injuries has reported an increase in BMD after 3 months of adjunctive acupuncture treatment, but the difference is not statistically significant (21).

Overall adjustment acupuncture (OA) was originally proposed by our research group based on our clinical exploration and experience for more than 20 years (22). OA combines the etiological and pathophysiological aspects of osteoporosis with kidney and spleen deficiency according to TCM theory, and it stimulates the meridian system, including the skin, collaterals, and meridians. OA has been shown to improve the clinical symptoms and quality of life in patients with osteoporosis (23). A systematic and comprehensive clinical study is also being conducted by our team to study the efficacy, safety, and mechanism of OA in patients with PMOP to provide more rigorous clinical evidence to promote its application (24). In the present study, we investigated the anti-osteoporotic effect of OA and explored its mechanism of action through endocrine modulation in a rat model of PMOP induced by bilateral ovariectomy.

## Materials and methods

### Animals

In total, 32 female Sprague–Dawley (SD) rats (aged 6 months old and weighing 250 ± 30 g) were purchased from the Experimental Animal Center of Chengdu Shuoda Bioscience Inc. (Chengdu, China). All animals were housed under environmentally controlled conditions (temperature 22 ± 3°C under a 12 h light/12 h dark cycle with lights on at 7:00) in polycarbonate cages with standard chow diet and water available ad libitum. All procedures were approved by the Ethics Committee of Yunnan University of Chinese Medicine (ethical code no. R-062021070). and performed in accordance with institutional guidelines.

### Establishment of animal model and treatment

**Figure 1:**
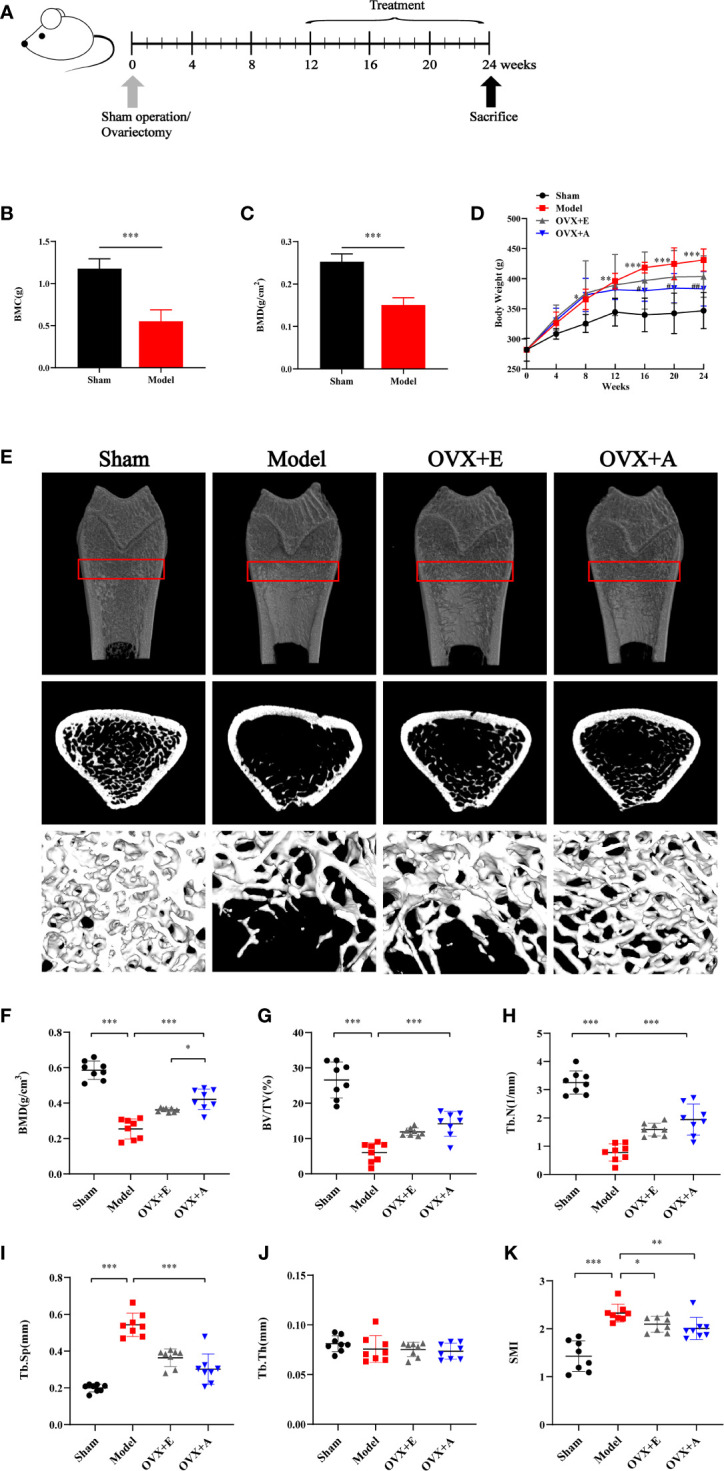
Experimental design and effects of OA and estradiol on body weight and bone tissue microstructure in OVX osteoporotic rats. **(A)** Ovariectomy (OVX) or sham operation was performed on 6-month-old rats. At 12 weeks postoperation after development of osteoporosis, acupuncture or estrogen treatment was implemented for 12 weeks. All rats were sacrificed at the end of week 24. **(B)** Bone mineral content (BMC). **(C)** Bone mineral density (BMD). **(D)** Body weight change. Data are presented as the mean ± SD (n=8). ** *P*<0.01 compared to the sham group; ***P < 0.001 compared to the sham group; # *P*<0.05 compared to the model group; ## *P*<0.01 compared to the model group. **(E)** Representative sample from each group showing the 2D mapping and 3D architecture of trabecular bone within the distal metaphyseal femur region. **(F)** Bone mineral density (BMD). **(G)** Ratio of trabecular volume/bone total volume. **(H)** Trabecular number (Tb.N). **(I)** Trabecular thickness (Tb.Th). **(J)** Trabecular separation (Tb.Sp). **(K)** Structure model index (SMI). Data are presented as the mean ± SD (n=8). * *P*<0.05, **P < 0.01, and ***P < 0.001.

After 1 week of acclimatization, all rats were randomly divided into the following four groups with eight rats in each group: sham, model, ovariectomy+estradiol (OVX+E), and OVX+OA (OVX+A). The rats in the model, OVX+E, and OVX+A groups were subjected to bilateral OVX, while rats in the sham group were subjected to sham operation (laparotomy and bilateral ovary exposal without resection). All rats were raised equally as previously described for 3 months after the operation to allow development of osteoporosis (25). At week 13 postoperation, the OVX+E group received estradiol valerate (Progynova^®^, Bayer, Diegem, Belgium) 0.2 mg/kg/i.g/qod for 12 weeks (26), while the sham and model groups were gavaged with an equal volume of saline. The OVX+A group received OA therapy without anesthetics. Briefly, after routine disinfection, acupuncture needles (Huanqiu acupuncture needles, 0.25 × 30 mm in size, stainless steel, Suzhou Acupuncture Supplies Co. Ltd., lot 200302) were inserted at Zusanli (ST36), ShenShu (BL23), and Dazhu (BL11) to a depth of 5–8 mm, manipulated with the twist compensation method to generate needling sensation and then maintained for 20 min. The selected acupoints were located according to the atlas of acupoints in rat as previously published (27). The OA treatment was conducted every other day and continued for 12 weeks (28, 29). The rats were kept conscious and secured as comfortably as possible during the treatment. The experimental scheme is shown in **Figure 1A**.

### Tissue sampling

At the end of the experiment, all rats were fasted overnight and weighed before sacrifice. Rats were anesthetized by an intraperitoneal injection of 2% sodium pentobarbital (50 mg/kg). Blood samples were collected by cardiac puncture and centrifuged at 3000 rpm for 10 min, and the serum was stored at −80°C. The uteri were immediately resected and weighed. The tibias and femurs were also immediately collected by removing the overlying soft tissues. The left tibiae were stored in normal saline at −20°C for subsequent DXA scanning. The left femurs were preserved in normal saline and sent for subsequent biomechanical assessment at 4°C. Femurs and tibias of the left limbs were preserved in 10% formalin/saline for subsequent histopathological examination.

### Micro-computed tomography analysis

Bone microarchitecture in the distal femur was scanned by micro-CT (Model ZKKS-MCT-Sharp). Briefly, the distal part of the femur was aligned perpendicularly to the scanning axis in a cylindrical sample holder for a total scanning length of 10 mm. The reconstruction and 3D quantitative analyses were performed by the desktop micro-CT system. The following 3D indices in the defined region of interest were analyzed: relative bone volume over total volume (BV/TV, %), trabecular separation (Tb.Sp), trabecular number (Tb.N), trabecular thickness (Tb.Th), structure model index (SMI), and BMD. The operator performing the scan analysis was blinded to the treatments.

### Three-point bending test

The left femurs were submitted to a three-point bending test for analysis of the biomechanical properties using a Servo-controlled tensile testing machine (High Speed Rail Testing Instruments Co. Ltd., Model AI-7000-M1, Dongguan, China). After thawing, the femoral neck was subjected to a bending-compression test. To achieve flexion-compression testing in the femora, the distal epiphysis was embedded in acrylic resin for fixing the base of the test machine. The load was applied in the center of the head, and the following parameters were used: bone load cell of 50 kgf, preload of 10.0 N, settling time of 30 s, and load application speed of 1 mm/min. The maximum load, maximum flexibility, elastic load, and elastic modulus of the femur were then calculated from the load/deformation curves.

### Hematoxylin and eosin and masson’s staining

The left femurs and tibias fixed in 10% neutral formalin for 72 h were immersed in 15% neutral EDTA buffer for 3 months for decalcification. The decalcified femurs were dehydrated with graded ethanol (50%–100%) in xylene, embedded in paraffin, sectioned at 5 μm thickness, and subjected to HE and Masson’s staining according to routine procedure (30, 31). After staining, the mounted slides were observed and photographed using an Olympus BX53 fluorescence microscope (Tokyo, Japan). The tissue sections were evaluated for microarchitectural changes, including the structure and morphology of trabecular bone and the trabecular area. The trabecular area of Masson’s staining was quantified using Image Pro Plus 6.0 software.

### Uterus index

The rat uterus was dissected, stripped of adipose tissue, and weighed on a precise electronic balance. The uterus wet weight was divided by the body weight of the rat to obtain the uterus index.

### Enzyme-linked immunosorbent assay

The serum levels of estradiol (E2) and bone turnover biomarkers, including alkaline phosphatase (ALP), procollagen type 1-N-peptide (PINP), serum C-terminal telopeptide of type 1 collagen (S-CTX), tartrate-resistant acid phosphatase (TRACP), corticotropin releasing hormone (CRH), adrenocorticotropic hormone (ACTH), and corticosterone (CORT), were determined by corresponding ELISA kits (Meimian, Jiangsu Meimian Industrial Co., Ltd.) according to the manufacturer’s instructions.

### Statistical analysis

Data are presented as the mean ± standard deviation (SD). Data were compared among groups using one-way analysis of variance (ANOVA) followed by Bonferroni’s test for pairwise comparisons between groups. Statistical analyses were performed using SPSS statistical software (version 20.0; IBM Corporation, Armonk, NY, USA). The significance level was set at *P*<0.05. The significant differences in figures are indicated by *P < 0.05, **P < 0.01, and *** *P* < 0.001.

## Results

### Effects of OA and estradiol on body weight and bone tissue microstructure in OVX osteoporotic rats

As shown in **Figure 1D**, the average weight of rats in each experimental group was similar at the start of the experiment, but more weight gain was observed in rats upon OVX compared to rats in the sham group during the experiment. However, the trend of weight gain in the OVX+E group and the OVX+A group decreased upon treatment, which started at week 13 and lasted until the end of the experiment (week 24). The mean body weight before sacrifice was significantly higher in the model group compared to the sham group (*P*=0.002) in week 12. However, the mean body weight was significantly lower in the OVX+A (*P*=0.002) groups compared to the model group in week 24. DXA was used to evaluate bone loss after the OVX operation. The BMC was significantly decreased in the model group compared to the sham group (*P*=9.313×10^-4^; **Figure 1B**). Similarly, the model group showed a significant decrease in BMD compared to the sham group (*P*=4.254×10^-4^; **Figure 1C**). Together, these results demonstrated establishment of a successful osteoporosis model.

Micro-CT was utilized to evaluate therapeutic effects of OA and estradiol treatment on bone loss of OVX osteoporotic rats respectively. Representative 2D and 3D micro-CT images of femurs as well as the bone morphologic parameters of rats from each group were presented in **Figure 1E**. Compared to the sham group, the model group showed significant bone loss and deterioration of bone structure (**Figure 1E**). In particular, the femoral head of rats in the model group clearly showed a porous architecture. However, treatment with estradiol or OA healed the OVX-induced trabecular damage, and the OVX+A group showed greater restoration of trabecular bone structure compared to the OVX+E group. Compared to the sham group, the BMD, BV/TV, Tb.N, and Tb.Th values were lower in the model group (*P=*7.747×10^-9^ for BMD; *P=*8.662×10^-7^ for BV/TV; *P=*1.631×10^-9^ for Tb.N; *P=*0.355 for Tb.Th, **Figures 1F–H, J**), and the Tb.Sp and SMI values were higher in the model group (*P=*7.566×10^-10^ for Tb.Sp; *P=*2.207×10^-5^ for SMI, **Figures 1I, K**). Compared to the model group, 12 weeks of estradiol or OA treatment reversed this situation. Moreover, a greater increase in BMD was observed in the OVX+A group than in OVX+E group (*P*=0.012; **Figure 1F**). Similarly, a greater increase in the BV/TV, Tb.N, and Tb.Th values as well as a greater decrease in the Tb.Sp and SMI values were observed in the OVX+A group compared to the OVX+E group, but no statistical significances were found. These results indicated that OA treatment significantly restored bone loss in OVX-induced osteoporotic rats.

### Effect of OA and estradiol treatment on femoral histomorphology in OVX osteoporotic rats

**Figure 2:**
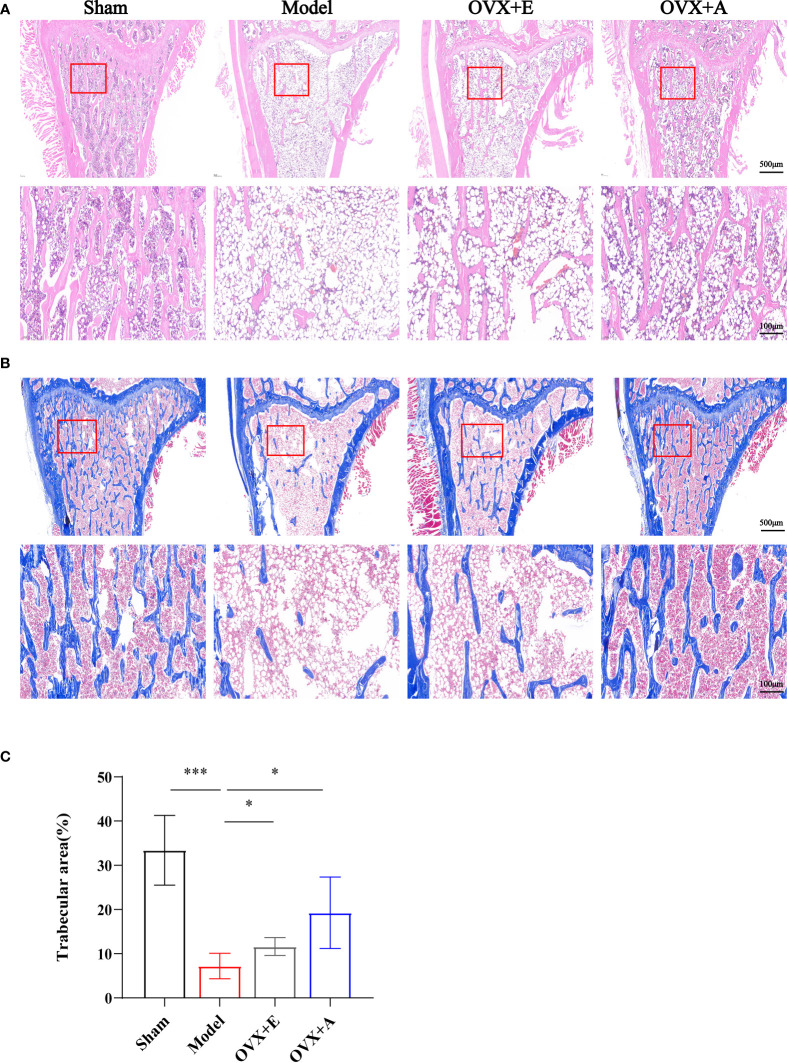
Effects of OA and estradiol treatment on femoral histomorphology in OVX osteoporotic rats. **(A)** Representative images of tissue sections of the metaphysis regions of distal femurs stained by hematoxylin-eosin (HE). The rectangle in the lower panel indicates the magnified areas. Scale bar: 500 μm in the upper panel and 100 μm in the lower panel. **(B)** Representative images of tissue sections of the proximal metaphysis regions of femurs by Masson’s staining. The rectangle in the lower panel indicates the magnified areas. Scale bar: 500 μm in the upper panel and 100 μm in the lower panel. **(C)** The percentages of trabecular areas were quantified using Image Pro Plus 6.0 software. Data are presented as the mean ± SD (n=4). *P < 0.05 and ***P < 0.001.

Representative histological images of the distal metaphyseal portion of the rat femurs stained with HE and Masson’s staining are shown in **Figure 2**. Compared to the sham group, the trabecular bone structure in the model group exhibited disrupted trabecular meshwork with thinner and irregular bone trabeculae and widened trabecular gaps, showing typical osteoporotic changes. In contrast, improved trabecular structure was observed in the metaphyseal portion of the distal femurs of rats in both the OVX+E and OVX+A groups with thicker trabeculae and narrowed trabecular gaps. Quantification of Masson-stained trabecular bone area using Image Pro Plus 6.0 software showed that the trabecular bone area was significantly lower in the model group compared to the sham group (*P*=7.846×10^-4^), However, the trabecular bone area was significantly higher in the OVX+E (*P*=0.046) and the OVX+A (*P*=0.030) groups compared to the model group. These results suggested that OA treatment ameliorated histomorphological changes and improved the microarchitecture of bone tissue in ovariectomized rats.

### Effect of OA and estradiol treatment on femoral biomechanical properties in OVX osteoporotic rats

**Figure 3:**
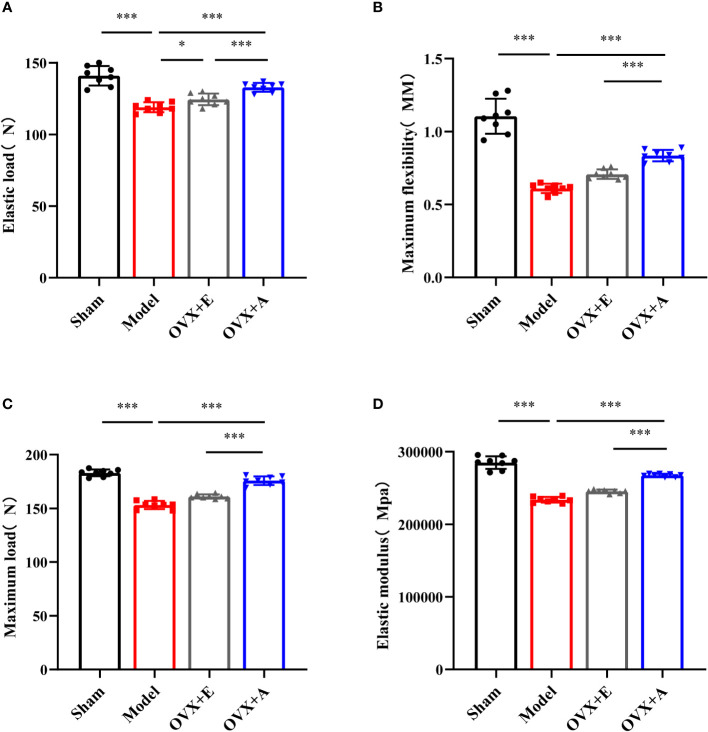
Effects of OA and estradiol treatment on the biomechanical properties of the femurs in OVX osteoporotic rats. **(A)** Maximum flexibility, **(B)** maximum load **(C)** and elastic modulus **(D)** of the femurs were measured by the three-point bending test. Data are presented as the mean ± SD (n=8). *P<0.05 and ***P<0.001.

The three-point bending test is one of the most frequently adopted techniques used to evaluate bone biomechanical properties (31). As shown in **Figure 3**, the strength of the femurs in the model group was significantly decreased compared to the sham group in terms of elastic load (*P=*1.134×10^-6^; **Figure 3A**), maximum flexibility (*P=*3.634×10^-6^; **Figure 3B**), maximum load (*P=*8.391×10^-11^; **Figure 3C**), and elastic modulus (*P=*4.416×10^-10^; **Figure 3D**). However, treatment with estradiol or OA partly restored bone strength in terms of these parameters, including elastic load (*P*=0.011 for estradiol; *P=*7.751×10^-7^ for OA), maximum flexibility (*P*=2.786×10^-5^ for estradiol; *P*=4.541×10^-9^ for OA), maximum load (*P*=1.262×10^-4^ for estradiol; *P*=1.414×10^-8^ for OA), and elastic modulus (*P*=3.742×10^-6^ for estradiol; *P*=3.283×10^-12^ for OA). Together, these results demonstrated that OA treatment improved the biomechanical properties of osteoporotic bone in ovariectomized rats.

### Effects of OA and estradiol treatment on uterine index, serum E2, bone turnover biomarkers and hypothalamic-pituitary-adrenal axis-related hormones in OVX osteoporotic rats

**Figure 4:**
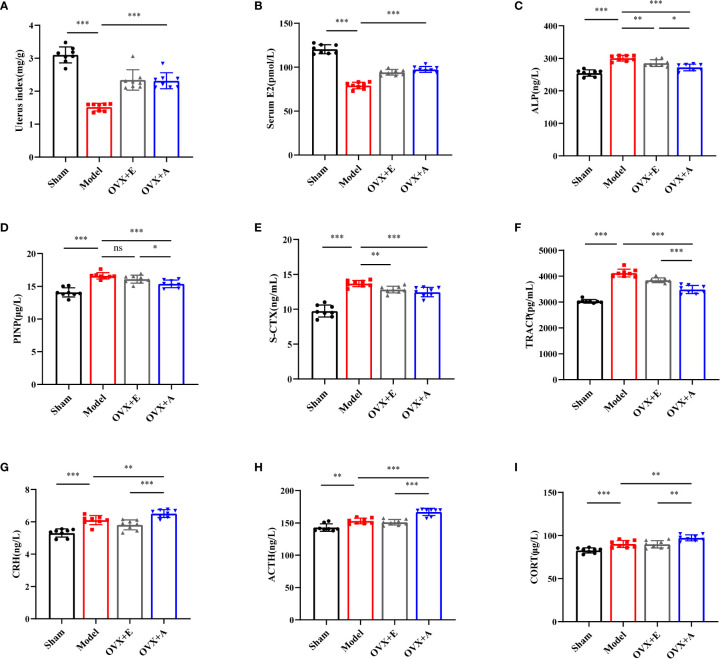
Effects of OA and estradiol treatment on uterine index, serum E2, bone turnover biomarkers and hypothalamic-pituitary-adrenal (HPA) axis-related hormones in OVX osteoporotic rats. **(A)** Uterus indexes of the experiment rats from each group. **(B)** Estradiol levels in the serum of rats from each group. Data are presented as the mean ± SD (n=8). ***P < 0.001. **(C)** Effects of OA on serum alkaline phosphatase (ALP). **(D)** Effects of estradiol and OA on serum propeptide of type I procollagen (PINP). **(E)** Effects of estradiol and OA on serum S-CTX. **(F)** Effects of estradiol and OA on serum TRACP. The values are presented as the mean ± SD (n=8). *P < 0.05, **P < 0.01, and ***P < 0.001. The serum levels of **(G)** corticotropin releasing hormone (CRH), **(H)** adrenocorticotropic hormone (ACTH), and **(I)** corticosterone (CORT) were measured by ELISA. Data are presented as the mean ± SD (n=8). **P < 0.01 and ***P < 0.001.

During sacrifice, overt atrophy and shrinkage of uteri were observed in the model group compared to the sham group. However, the uteri in the OVX+A group appeared larger than those in the model group. An increase in the volume of the uteri was also observed in the OVX+E group compared to the model group. Because comparison of the uterus indexes (**Figure 4A**) coincided with the macroscopic observation, we investigated the systemic estrogen level of experiment rats. The serum estradiol levels were significantly lower in the model group compared to the sham group (*P*=3.473×10^-11^; **Figure 4B**). However, the estradiol levels were significantly restored in both the OVX+A group and the OVX+E group compared to the model group (*P*=5.789×10^-7^ for estradiol; *P*=5.853×10^-8^ for OA). Thus, these results demonstrated that OA treatment modulated the systemic estrogen level in ovariectomized rats.

To explore whether OA combined with alendronate treatment plays a role in bone metabolism, we evaluated serum bone turnover markers in OVX rats. The serum bone turnover markers, including ALP (*P*=1.188×10^-7^; **Figure 4C**), PINP (*P*=8.305×10^-7^, **Figure 4D**), S-CTX (*P*=1.496×10^-8^; **Figure 4E**), and TRACP (*P*=2.763×10^-11^; **Figure 4F**), were significantly increased in the model group compared to the sham group. However, treatment with estradiol or OA significantly restored the serum levels of the bone turnover markers, including ALP (*P*=0.006 for estradiol; *P*=3.057×10^-5^ for OA), PINP (*P*=0.086 for estradiol; *P*=4.420×10^-4^ for OA), S-CTX (*P*=0.002 for estradiol; *P*=8.482×10^-4^ for OA), and TRACP (*P*=5.310×10^-4^ or estradiol; *P*=9.372×10^-7^ for OA), to levels similar to those in the sham group. Together, these results showed that OA treatment suppressed OVX-induced bone turnover.

To further investigate the endocrine-modulating effect of OA, activation of the HPA axis was investigated by measuring the serum levels of CRH, ACTH, and CORT by ELISA. Compared to the sham group, the CRH (*P*=3.978×10^-5^; **Figure 4G**), ACTH (*P*=0.001; **Figure 4H**), and CORT (*P*=8.248×10^-4^; **Figure 4I**) levels were significantly increased in the model group. Moreover, a further increase in CRH (*P*=0.008 for OA), ACTH (*P*=6.697×10^-5^ for OA), and CORT (*P*=0.002 for OA) was observed in the OVX+A group compared to the model group. However, no statistical differences were observed in the HPA hormones in the OVX+E group compared to the model group. These results suggested that OA exerted a modulatory effect on the activity of the HPA axis in ovariectomized rats.

## Discussion

In the present study, we demonstrated that OA improved osteoporosis in terms of BMD, BMC, bone tissue biomechanical properties, and bone tissue histomorphology. We also demonstrated that OA increased systemic estrogen levels and that OA modulated the HPA axis function in ovariectomized rats. Beneficial effects of acupuncture on osteoporosis in ovariectomized animals have been previously reported, demonstrating improvements in BMD, bone strength, bone structure, and bone turnover (28, 29, 32, 33). Consistent with these results, the present study demonstrated that OA improved osteoporosis in the OVX rat model despite differences in types of acupuncture and specificity of acupoint selection.

The present study demonstrated that OA increased the uterus index and serum estrogen levels in the OVX rat model, which agreed with previous studies on acupuncture treatment of ovariectomized animals (34, 35). Moreover, the efficacy of acupuncture has been reported to reverse osteoporosis in the SAMP6 mouse model of senile osteoporosis by enhancing the secretion of testosterone and decreasing bone turnover (36). These studies suggest a regulatory effect of acupuncture on hormonal profiles, and the improvement of PMOP by acupuncture may be closely related to this modulatory effect of sex hormone levels. However, these results were obtained from studies on non-human subjects, and evidence in human subjects is lacking. Our ongoing clinical trial was designed to collect data, including estradiol levels, in patients with PMOP upon OA treatment to cover this gap (24).

In postmenopausal women, the hypothalamic-adrenal-ovarian (HPO) axis tends to function at a low level and is accompanied by a significant decrease in ovarian-derived estrogen levels. Estrogen is then synthesized in extraglandular sites (a process named extragonadal aromatization), such as adipose tissue, liver, muscle, and several parts of the brain, thus providing a pathway for target tissues to synthesize and metabolize estrogen according to local needs (37–39). This process requires the involvement of a microsomal member of the cytochrome P450 superfamily, the aromatase cytochrome P450 (P450arom) (40). Aromatase catalyzes the demethylation of carbon 19 of androgen to produce phenolic 18-carbon estrogens (41). It has been reported that acupuncture promotes extragonadal aromatization and increases blood estrogen concentrations in OVX rats due to the contribution of abdominal subcutaneous fat and liver tissue (42). However, as the expression levels of aromatase vary among tissue types, the biosynthesis and function of estrogen are regulated locally in a paracrine or intracrine manner within individual tissue. The regulation of this process is unique in each target tissue, and circulating synthetic estrogens may also affect local metabolism, leading to a complex physiological situation that makes the interpretation of circulating estrogen levels more difficult (40, 43). To date, few studies have reported the aromatization in bone tissue upon acupuncture, and our future study aims will focus on this aspect.

C19-steroids, synthetic estrogen precursors, are produced mainly by adrenal glands (44, 45). Dehydroepiandrosterone (DHEA) and its sulfate (DHEA-S), which are secreted by the adrenal glands, are converted to androstenedione (ASD) and then to androgens and estrogens in peripheral tissues, thus providing autonomous production and metabolism of sex steroids according to local needs (46). In postmenopausal women, estrogen is almost exclusively synthesized locally in peripheral target tissues from inactive adrenal C19-steroid precursors secreted by the adrenal cortex, particularly DHEA (47). In addition, the circulating levels of adrenal C19-steroids may directly reflect the parameters of adrenal sex precursor steroid secretion levels (48). In a previous trial reporting a 12-month evaluation of the efficacy of DHEA replacement therapy in 14 women aged 60–70 years who used 10% DHEA cream daily, hip BMD increased in the absence of stimulatory effects on the endometrium, which correlated with a 20% reduction in plasma bone alkaline phosphatase and a 28% reduction in the urinary hydroxyproline/creatinine ratio (49).

Because the HPA axis is responsible for stimulating adrenal corticosteroids in response to changes and stresses in the body, we investigated whether OA also exerts a modulatory effect on HPA axis hormone secretion in the OVX rat model by measuring the serum levels of CRH, ACTH, and CORT. Similar to the results of our study, Zhao et al. reported that electroacupuncture increases the release of CRH from the paraventricular nucleus and increases blood estrogen levels in OVX rats. By comparing the effects of electroacupuncture and natural substitution on OVX rats, a previous study has reported that electroacupuncture may accelerate the process of endogenous substitution of estrogen synthesis (50, 51). Chen et al. reported a stimulatory effect of electroacupuncture on nucleolar organizer regions (NORs) of the adrenal cortex in ovariectomized rats, and they reported that the vaginal smears showed a response similar to estrogen-induced response with the appearance of exfoliative cells and that this response was not observed in normal rats upon electroacupuncture treatment, suggesting that electroacupuncture may promote the synthesis and secretion of adrenal steroid hormones. The produced androgen will then be transformed into estrogen in other tissues, thus compensating the deficiency of estrogen induced by ovariectomy (52).

In general, stress is considered to be an effective stimulus for HPA axis activity and CORT secretion. In addition, CORT is often referred to as the stress hormone. However, HPA axis research suggests that it may not be feasible to simply equate elevated CORT with stress. For example, some conditions that are not usually considered stressors, such as exercise and anti-anxiety medications, also cause CORT secretion; whereas some conditions that are usually considered stressful, such as chronic neuropathic pain, are not necessarily associated with acute CORT secretion (53, 54). More specifically, in the presence of a hypofunctional HPO axis, the HPA axis is an important bridge for the coordination of the central nervous system and neuroendocrine systems with each other due to its activation of endogenous adrenal compensatory effects and potential role in maintaining dynamic homeostasis (38, 40). Importantly, acupuncture, as a benign regulator, may accelerate this ability to regain dynamic balance (51).

Acupuncture improves osteoporosis while increasing circulating estrogen levels, which may be closely related to postmenopausal extragonadal aromatization. It has been shown that chondrocytes and osteoblasts of bone also have the ability to express aromatase and thus can locally synthesize estrogen (55). In particular, the risk of osteoporosis and the incidence of fractures are increased for patients treated with aromatase inhibitors for breast cancer (56, 57). Therefore, the pathway of acupuncture stimulation to activate extragonadal aromatization still needs to be further explored.

In conclusion, the present study demonstrated that OA improves osteoporosis and exerts a modulatory effect on systemic estrogen levels and HPA axis activity in OVX rats. Further studies are needed to determine whether OA acts through increasing the aromatization in bone tissue locally or increasing estrogen produced by aromatization in other tissues as well as to determine how OA regulates bone metabolism.

## Data availability statement

The original contributions presented in the study are included in the article/supplementary material. Further inquiries can be directed to the corresponding authors.

## Ethics statement

The animal study was reviewed and approved by The Ethics Committee of Yunnan University of Chinese Medicine (ethical code no. R-062021070).

## Author contributions

All authors listed have made a substantial, direct, and intellectual contribution to the work and approved it for publication.

## Funding

This study was supported by the National Natural Science Foundation of China (81560799, 82060895, 31960178, 82160923); Applied Basic Research Programs of Science and Technology Commission Foundation of Yunnan Province (2019FA007); Key Laboratory of Traditional Chinese Medicine for Prevention and Treatment of Neuropsychiatric Diseases, Yunnan Provincial Department of Education; Scientific Research Projects for High-level Talents of Yunnan University of Chinese Medicine (2019YZG01); Young Top-Notch Talent in 10,000 Talent Program of Yunnan Province (YNWR-QNBJ-2019-235); National Science and Technology Innovation 2030 Major Program (2021ZD0200900); Yunnan Key Research and Development Program (202103AC100005); Yunnan Province Fabao Gao Expert Workstation Construction Project (202105AF150037).

## Acknowledgments

We would like to express our sincere gratitude to all those who participated in this study or helped with this study.

## Conflict of interest

The authors declare that the research was conducted in the absence of any commercial or financial relationships that could be construed as a potential conflict of interest.

## Publisher’s note

All claims expressed in this article are solely those of the authors and do not necessarily represent those of their affiliated organizations, or those of the publisher, the editors and the reviewers. Any product that may be evaluated in this article, or claim that may be made by its manufacturer, is not guaranteed or endorsed by the publisher.
